# Supplementary material for: The Association of Bisphenol A and Phthalates with Risk of Breast Cancer: A Meta-Analysis
Source: Int J Environ Res Public Health. 2021 Mar 1;18(5):2375. doi: 10.3390/ijerph18052375 (PMC7967730; doi:10.3390/ijerph18052375)
Supplement: Supplementary file 1 [file ijerph-18-02375-s001.pdf]

The association of bisphenol A and phthalates with risk of breast cancer：A meta-analysis

Table S1. Quality assessment of the included studies by Newcastle-Ottawa standard.

Study	Selection				Comparability	Exposure			Score
	Q1	Q2	Q3	Q4	Q5	Q6	Q7	Q8	
K W. Reeves (2018)	*	*	-	*	*	*	*	-	6
M Morgan (2017)	-	-	*	*	*	*	*	-	5
A Aschengrau (1998)	*	*	*	*	* *	*	*	-	8
B Traber (2014)	*	*	*	*	*	*	*	-	7
H Parada (2020)	*	*	*	*	*	*	*	-	7
A K. Holmes （2014）	*	*	-	*	*	*	*	-	6
L. López-Carrill (2010)	*	*	*	*	* *	*	*	-	8

K W. Reeves (2019)	*	*	-	*	*	*	*	-	6
H Parada (2018)	*	*	*	*	*	*	*	-	7

Quality assessment of case-control studies

Selection

Q1: Is the case definition adequate?

a) yes, with independent validation *

b) yes, eg record linkage or based on self-reports

c) no description

Q2: Representativeness of the cases

a) consecutive or obviously representative series of cases *

b) potential for selection biases or not stated

Q3: Selection of Controls

a) community controls *

b) hospital controls

c) no description

Q4: Definition of Controls

a) no history of disease (endpoint) *

b) no description of source

Comparability

Q5: Comparability of cases and controls on the basis of the design or analysis

a) study controls for age *

b) study controls for any additional factor *

Exposure

Q6: Ascertainment of exposure

a) secure record *

b) structured interview where blind to case/control status *

c) interview not blinded to case/control status

d) written self-report or medical record only

e) no description

Q7: Same method of ascertainment for cases and controls

a) yes *

b) no

Q8: Non-Response rate

a) same rate for both groups *

b) non respondents described

c) rate different and no designation

Table S2. Results of sensitivity analyses by removing each study.

Study Omitted	Meta-analyses	Heterogeneity	
	OR (95% CIs)	I^2 (%)	p value
**Association between BPA and breast cancer risk**			
K W. Reeves (2018)	0.85(0.68-1.06)	0.0	0.542
M Morgan (2017)	0.87(0.69-1.10)	0.0	0.582
A Aschengrau (1998)	0.86(0.69-1.09)	0.0	0.553
B Traber (2014)	0.78(0.61-0.99)	0.0	0.980
H Parada (2020)	0.91(0.70-1.18)	0.0	0.692
Combined	0.85(0.69-1.05)	0.0	0.705
**Association between MBzP and breast cancer risk**			
A K. Holmes (2014)	0.69(0.56-0.85)	0.0	0.602
M Morgan (2017)	0.73(0.59-0.91)	41.6	0.144
A. Aschengrau (1998)	0.79(0.63-0.98)	0.0	0.477
K W. Reeves (2019)	0.72(0.57-0.92)	41.1	0.148
H Parada (2018)	0.74(0.58-0.94)	41.5	0.145
Combined	0.73(0.60-0.90)	27.0	0.232
**Association between MEP and breast cancer risk**			
A K. Holmes (2014)	1.06(0.66-1.69)	75.8	0.006
M Morgan (2017)	1.06(0.65-1.73)	74.9	0.007
L. López-Carrillo (2010)	0.83(0.65-1.05)	0.9	0.388

K W. Reeves (2019)	0.93(0.52-1.67)	79.9	0.002
H Parada (2018)	0.97(0.53-1.77)	79.1	0.002
Combined	0.96(0.62-1.48)	73.2	0.001
**Association between MEHHP and breast cancer risk**			
A K. Holmes (2014)	1.08(0.84-1.39)	0.0	0.423
M Morgan (2017)	1.11(0.84-1.45)	15.4	0.307
L. López-Carrillo (2010)	1.05(0.80-1.38)	0.0	0.467
H Parada (2018)	1.30(0.95-1.77)	0.0	0.806
Combined	1.12(0.88-1.42)	0.0	0.497
**Association between urinary MEHP and breast cancer risk**			
A K. Holmes (2014)	0.93(0.71-1.20)	1.0	0.364
M Morgan (2017)	1.23(0.69-2.18)	72.7	0.026
L. López-Carrillo (2010)	1.13(0.61-2.08)	70.2	0.035
H Parada (2018)	1.34(0.82-2.18)	45.7	0.159
Combined	1.13(0.74-1.73)	59.7	0.059
**Association between urinary MEOHP and breast cancer risk**			
A K. Holmes (2014)	0.99(0.70-1.40)	0.0	0.333
M Morgan (2017)	0.92(0.61-1.38)	0.0	0.491
L. López-Carrillo (2010)	1.18(0.77-1.80)	0.0	0.942
Combined	1.01(0.74-1.40)	0.0	0.587
**Association between urinary MCPP and breast cancer risk**			
L. López-Carrillo (2010)	0.89(0.63-1.25)	70.9	0.064
K W. Reeves (2019)	0.60(0.38-0.94)	43.9	0.182
H Parada (2018)	0.71(0.31-1.60)	86.1	0.007

Combined	0.80(0.41-1.56)	83.8	0.002
**Association between urinary MBP and breast cancer risk**			
A K. Holmes (2014)	0.85(0.56-1.30)	0.0	1.000
M Morgan (2017)	0.77(0.48-1.22)	0.0	0.602
L. López-Carrillo (2010)	0.77(0.48-1.22)	0.0	0.600
Combined	0.80(0.55-1.15)	0.0	0.843
**Association between urinary MiBP and breast cancer risk**			
M Morgan (2017)	0.77(0.57-1.04)	0.0	0.809
L. López-Carrillo (2010)	0.76(0.57-1.03)	0.0	0.740
H Parada (2018)	0.72(0.50-1.04)	0.0	0.941
Combined	0.75(0.58-0.98)	0.0	0.937
